# Measuring constructs from the Consolidated Framework for Implementation Research in the context of increasing colorectal cancer screening at community health centers

**DOI:** 10.1186/1748-5908-10-S1-A10

**Published:** 2015-08-14

**Authors:** Shuting Liang, Michelle Kegler, Michelle Carvalho, Maria Fernandez, Bryan Weiner, Sara Jacobs, Rebecca Williams, Betsy Risendal, Letoynia Coombs, Daniela Friedman, Beth Glenn, Shin-Ping Tu

**Affiliations:** Emory Prevention Research Center, Department of Behavioral Sciences and Health Education, Rollins School of Public Health, Emory University, Atlanta, GA 30322 USA; School of Public Health, University of Texas Health Science Center at Houston, Houston, TX 77030 USA; Department of Health Policy and Management, UNC Gillings School of Global Public Health, University of North Carolina at Chapel Hill, Chapel Hill, NC 27599 USA; Public Health Research Division, RTI International, Research Triangle Park, NC, 27709 USA; Lineberger Comprehensive Cancer Center, School of Medicine, University of North Carolina at Chapel Hill, Chapel Hill, NC 27599 USA; University of Colorado Comprehensive Cancer Center, Aurora, CO 80045 USA; Department of Community and Behavioral Health, Colorado School of Public Health, Aurora, CO 80045 USA; Department of Health Promotion, Education, and Behavior, Arnold School of Public Health, University of South Carolina, Columbia, SC 29208 USA; Statewide Cancer Prevention and Control Program, Arnold School of Public Health, University of South Carolina, Columbia, SC 29208 USA; UCLA Kaiser Permanente Center for Health Equity, University of California Los Angeles, Los Angeles, CA 90095-6900 USA; Fielding School of Public Health, University of California Los Angeles, Los Angeles, CA 90095 USA; Jonsson Comprehensive Cancer Center, University of California Los Angeles, Los Angeles, CA 90095 USA; Department of Medicine, Virginia Commonwealth University, Richmond, VA 23298 USA

## Background

The Consolidated Framework for Implementation Research (CFIR) is a comprehensive meta-framework widely applied to implementation related studies. Yet, few have used validated measures to operationalize constructs in CFIR in real-life settings. In this study, we operationalized selected CFIR constructs in an assessment to identify factors influencing implementation of evidence-based practices for increasing colorectal cancer screening in Community Health Centers (CHC).

## Methods

We selected 16 constructs from all five domains of CFIR. Measures were developed and tested in a cross-sectional survey with CHCs' clinical staff and leaders respectively. We performed a separate confirmatory factor analysis (CFA) for measures with three or more items, computed inter-item consistency (Cronbach's alpha), inter-rater reliability (ICC) and agreement (rWG(J)) statistics, and assessed construct validity via inter-correlations among constructs at individual and organizational levels.

## Results

A total of 277 individuals and 59 CHC clinics were included in the analysis. CFA showed satisfactory structural validity (CFI>0.90, TLI>0.90, SRMR<0.08, RMSEA<0.08); all measures showed reasonable reliability (alpha>0.70). The ICCs (>0.1) and rWG(J) (>0.75) suggest it appropriate to aggregate individual responses by computing clinic means. Results also suggest good construct validity at both individual and clinic levels. Inner setting and process-related constructs are correlated with most variables across domains; correlations between outer setting and intervention characteristics and other domains vary more noticeably by construct.

## Implications

Our study is one of the first to quantitatively measure constructs from all five domains of CFIR and demonstrate their psychometric properties. We depicted their inter-correlations at multiple levels, which set the foundation for establishing predictive models, causal pathways and developing interventions that target these factors. These findings could contribute to further development of the CFIR.

